# Network analysis of posttraumatic stress and posttraumatic growth symptoms among women in subsequent pregnancies following pregnancy loss

**DOI:** 10.1186/s12888-024-05702-6

**Published:** 2024-04-09

**Authors:** Qiaoqiao Shen, Qi Fu, Chen Mao

**Affiliations:** 1 Department of Epidemiology, School of Public Health, Southern Medical University, 510515 Guangzhou, Guangdong China; 2 School of Nursing, Southern Medical University, Guangzhou, Guangdong China

**Keywords:** Posttraumatic stress, Posttraumatic growth, Pregnancy loss, Pregnant woman, Network analysis

## Abstract

**Background:**

Pregnant women who have undergone pregnancy loss often display both posttraumatic stress (PTS) and posttraumatic growth (PTG). However, the precise relationship and structure of symptomatic levels of PTS and PTG have not been well understood. This study aimed to assess the associations between PTS and PTG symptoms in women during subsequent pregnancies following a previous pregnancy loss.

**Methods:**

A total of 406 pregnant women with a history of pregnancy loss were included in this study. The Impact of Events Scale-6 (IES-6) and the Posttraumatic Growth Inventory Short Form (PTGI-SF) were used to assess symptoms of PTS and PTG, respectively. The Graphical Gaussian Model was employed to estimate the network model. Central symptoms and bridge symptoms were identified based on “expected influence” and “bridge expected influence” indices, respectively. The stability and accuracy of the network were examined using the case-dropping procedure and nonparametric bootstrapped procedure.

**Results:**

The network analysis identified PTG3 (“Ability to do better things”) as the most central symptom, followed by PTS3 (“Avoidance of thoughts”) and PTG6 (“New path for life”) in the sample. Additionally, PTS3 (“Avoidance of thoughts”) and PTG9 (“Perception of greater personal strength”) were bridge symptoms linking PTS and PTG clusters. The network structure was robust in stability and accuracy tests.

**Conclusions:**

Interventions targeting the central symptoms identified, along with key bridge symptoms, have the potential to alleviate the severity of PTS experienced by women with a history of pregnancy loss and promote their personal growth.

**Supplementary Information:**

The online version contains supplementary material available at 10.1186/s12888-024-05702-6.

## Background

Pregnancy loss, also known as the unintentional demise of the fetus before reaching viability or the termination of pregnancy due to medical reasons [1], is a traumatic event that profoundly impacts a significant number of women worldwide [2]. For many women, especially those aspiring to conceive, it is likely that they will become pregnant again within 1–2 years following a pregnancy loss [3]. Throughout subsequent pregnancies, women may grapple with conflicting emotions and psychological phenomena [4–6]. On one hand, the previous pregnancy loss casts a shadow over them, resurfacing with the new pregnancy [4, 5]. They may find themselves revisiting past pregnancy experiences repeatedly, questioning their efforts, and experiencing feelings of guilt or remorse for the lost child [4, 5]. On the other hand, through the process of self-adjustment, women may gradually come to accept and confront the reality of pregnancy loss [6]. The new pregnancy can provide an opportunity for them to regain confidence in fertility and contribute to reshaping a positive mindset and emotional connection [7].

Previous research has indicated that women who experience pregnancy loss may manifest both posttraumatic stress (PTS) and posttraumatic growth (PTG) symptoms [8, 9]. However, there is a lack of quantitative studies focusing on the concurrent presence of PTS and PTG in this specific demographic, particularly during subsequent pregnancies. Research conducted among other populations indicates that the correlation direction between PTS total scores and PTG total scores does not always remain consistent, showing positive correlations, negative correlations, and curvilinear relationships [10]. In recent times, scholars have proposed that examining the relationship between PTS and PTG solely from a holistic perspective may overlook their interaction at symptom levels [11, 12]. As a result, some studies have attempted to utilize network analysis techniques to explore central symptoms and bridge symptoms in PTS and PTG networks, enhancing the understanding of the underlying psychopathological mechanisms associated with PTS and PTG [13–17].

Given the considerable heterogeneity in responses to various types of trauma among different populations [18], it is challenging to apply existing co-occurrence networks of PTS and PTG to pregnant women who have experienced pregnancy loss. Therefore, this study aimed to utilize network analysis to explore the interaction between PTS and PTG symptoms during early pregnancy in women who have undergone pregnancy loss, with the objective of establishing a theoretical foundation for future interventions by identifying crucial nodes with cascading effects within the network.

## Methods

### Participants

This was a multicenter, cross-sectional study conducted in Guangdong Province, China, from October 8, 2022, to July 20, 2023. Clinical staff recruited participants on-site, and upon obtaining informed consent from eligible individuals, they promptly distributed electronic versions of the survey questionnaire. The inclusion criteria for participants were as follows: (1) aged 18 years or older, (2) in the first trimester of pregnancy, (3) having a single intrauterine pregnancy, (4) having experienced miscarriage, stillbirth, or termination for medical reasons, and (5) expressing willingness to participate in this study. Individuals diagnosed with severe pregnancy complications, a history of psychological disorders, or those currently receiving psychological therapy were excluded. Ultimately, 379 pregnant women from three tertiary hospitals and 94 from a community hospital were invited to participate. A total of 406 individuals successfully completed the survey questionnaire, resulting in an effective response rate of 85.8%.

### Measurement

#### Sociodemographic characteristics

Participants self-reported their information, providing details on age, educational background, monthly household income, marital status, number of children, current conception method (natural conception or assisted reproductive technology), pregnancy complications, as well as the number and types of prior pregnancy losses.

### PTS symptoms

The Impact of Events Scale-6 (IES-6) [19] was utilized to assess PTS symptoms related to a previous pregnancy loss. The IES-6 comprises three subscales: intrusion, avoidance, and hyperarousal, each consisting of two items. Each item is rated on a scale from 0 (not at all) to 4 (extremely), resulting in a total score ranging from 0 to 24. A total score of 10 or higher indicates the presence of posttraumatic stress disorder (PTSD) [19]. The IES-6 has been widely used among Chinese populations [20], and it demonstrated good reliability and validity in this study.

### PTG symptoms

The Posttraumatic Growth Inventory Short Form (PTGI-SF) [21] was utilized to assess the manifestations of personal growth in women who had experienced pregnancy loss. The PTGI-SF comprises five dimensions: interpersonal relationships, personal strength, new possibilities, spiritual change, and appreciation of life, totaling 10 items. Each item is rated on a scale from 0 (no change) to 5 (complete change). The overall score ranges from 0 to 50, with higher scores indicating a greater level of PTG. The PTGI-SF is the most commonly used tool for measuring PTG [15], and it demonstrated acceptable reliability and validity in this study.

### Statistical analysis

All analyses were conducted using R software (Version 4.2.3). Continuous variables were described as mean (standard deviation, SD), and categorical variables were presented as frequencies and percentages.

We computed polychoric correlations between all nodes to examine the edges of the network and estimated the Graphical Gaussian Model (GGM) using the graphical least absolute shrinkage and selection operator (LASSO) in combination with the Extended Bayesian Information Criterion (EBIC) model [22]. In the network model, each symptom is represented as a “node,” and the association between symptoms is defined as an “edge.” As the association between PTS and PTG symptoms can be either positive or negative, we utilized the expected influence (EI) to quantify the impact of each node in the network. Nodes with high EI values can activate other nodes within the network, making them essential components of their own network [11]. To identify bridge nodes that connect PTS and PTG, we calculated the bridge expected influence (BEI). A higher positive value of the BEI for a node indicates a greater activation capacity towards nodes in another cluster, whereas a higher negative value signifies a stronger deactivation capacity towards nodes in another cluster [11, 23].

Additionally, we conducted verification of network accuracy and stability [22]. First, centrality stability was evaluated through a case-drop bootstrap procedure, whereby centrality indices were repeatedly computed from subsets of data with an increasing proportion of cases removed. A correlation stability (CS) coefficient value above 0.5 indicates a high level of stability for centrality indices of nodes within the network. Second, edge weight accuracy was estimated with bootstrapped 95% confidence intervals (CIs) by resampling the data 1,000 times. Finally, bootstrapped difference tests (α = 0.05) were conducted to determine if there were significant differences among edge weights and node EIs.

## Results

### Characteristics of the participants

The study involved participants with an average age of 30.93 years (SD = 4.76). Out of the 406 participants, 296 (72.9%) reported experiencing a single pregnancy loss, while 110 (27.1%) reported having undergone recurrent pregnancy losses (two or more losses). Concerning the current pregnancy, the majority (89.4%) occurred through natural conception, while 43 participants utilized assisted reproductive technology. Further demographic details of the participants can be found in Table 1. The average score for IES-6 was 6.64 (SD = 3.65), and 14.3% of participants scored above the cutoff point of 10, suggesting the presence of post-traumatic stress disorder (PTSD). The mean score for PTGI-SF was 32.29 (SD = 8.90). The mean and standard deviation for both IES-6 and PTGI-SF items can be found in Supplementary Table S1.

**Table 1:** Summary of participants characteristics (*N* = 406)

Variables	N	%
**Age**		
< 35 years	319	78.6
≥ 35 years	87	21.4
**Educational background**		
With a college/university degree	153	37.7
Without a college/university degree	253	62.3
**Monthly household income per capita**		
≤ ¥5000	109	26.8
¥5001–10,000	171	42.1
¥10,001–15,000	76	18.7
≥ ¥15,001	50	12.3
**Marital status**		
Married	374	92.1
Other	32	7.9
**Number of children**		
0	189	46.6
1	176	43.3
≥ 2	41	10.1
**Method of conception**		
Natural conception	363	89.4
Assisted reproductive technology	43	10.6
**Pregnancy complications**		
Yes	26	6.4
No	380	93.6
**Number of pregnancy loss**		
1	296	72.9
≥ 2	110	27.1
**Types of pregnancy loss**		
Miscarriage	336	82.8
Stillbirth	24	5.9
Termination for medical reasons	46	11.3

### Network structure

**Fig. 1:**
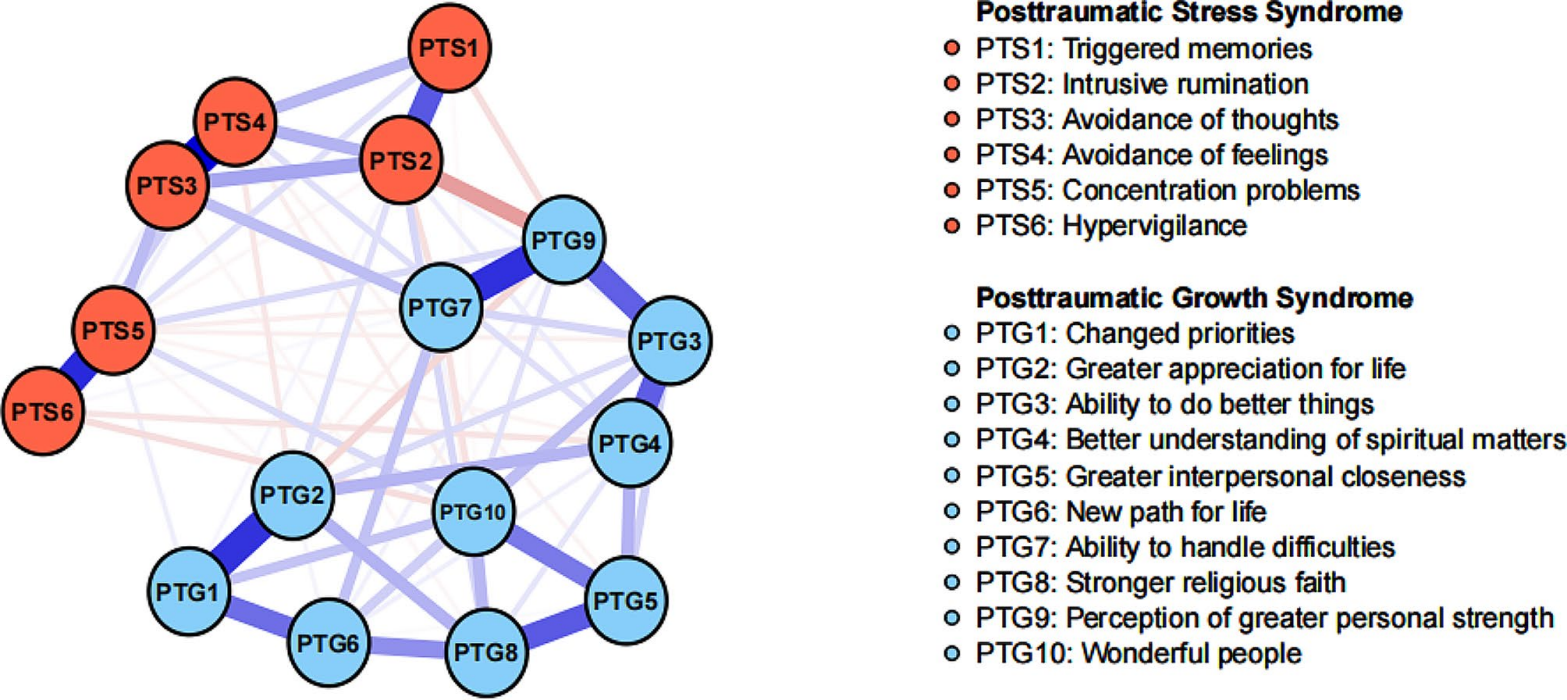
The network structure of PTS and PTG symptoms Nodes: Blue edges represent positive associations, and red edges indicate negative associations. Thicker edges reflect stronger associations

Figure 1 illustrates the network structure of the co-occurrence of PTS and PTG. The network exhibited a high density (0.72) with an average weight of 0.06. The PTS and PTG symptoms formed distinct clusters, with a few modest connections between them. Among the 86 non-zero edges connecting 16 nodes, 58 were positive edges, and 28 were negative edges. The strongest positive edge was found between PTS3 (“Avoidance of thoughts”) and PTS4 (“Avoidance of feelings”) (Edge weight value: 0.56), while the strongest negative edge was observed between PTG9 (“Perception of greater personal strength”) and PTS2 (“Intrusive rumination”) (Edge weight value: -0.25). The correlation matrix for PTS and PTG items is presented in Supplementary Table S2.

**Fig. 2:**
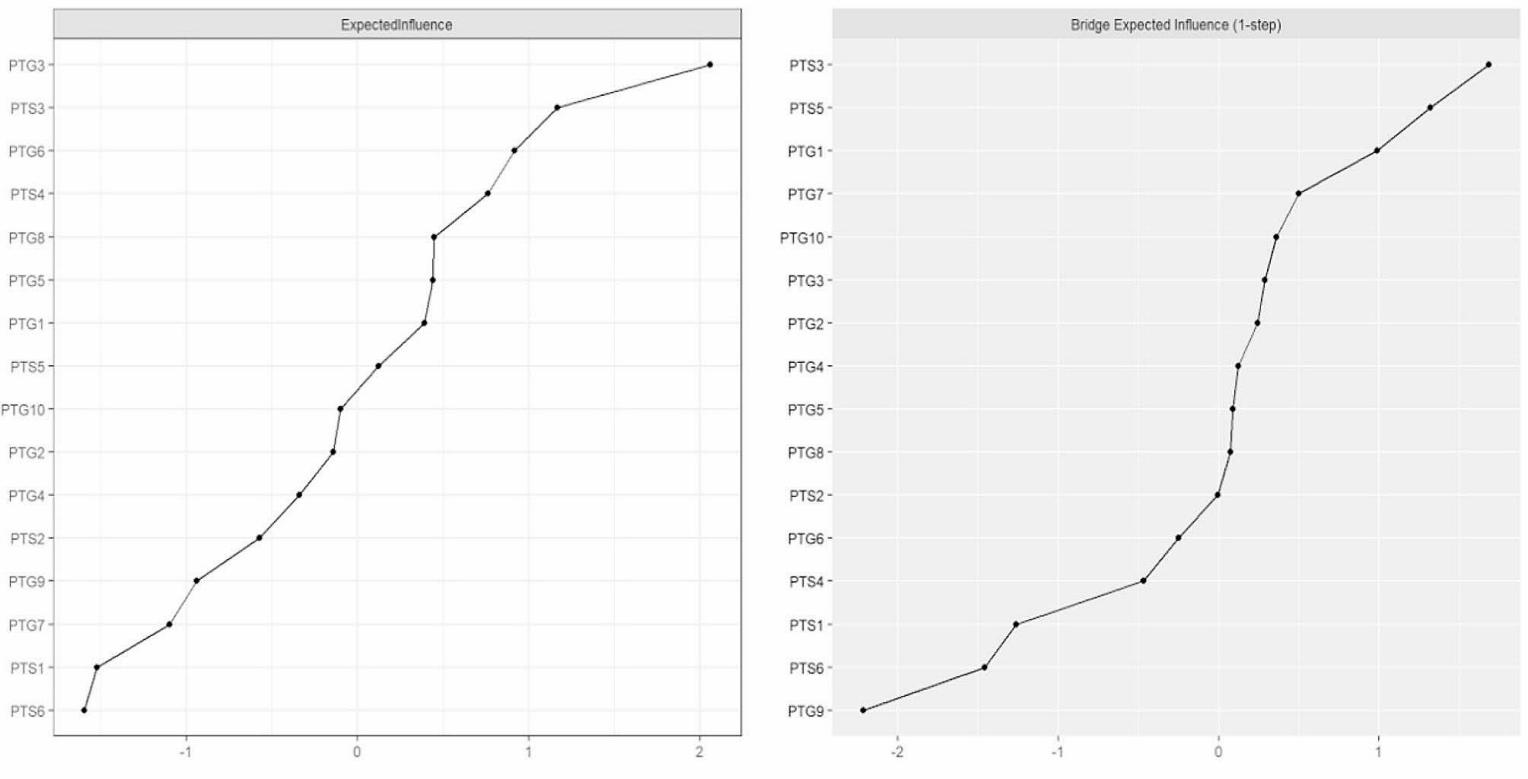
Expected influence and bridge expected influence of PTS and PTG symptoms

In terms of EI centrality, the node PTG3 (“Ability to do better things”) had the highest EI value (2.06), followed by PTS3 (“Avoidance of thoughts”) (EI value: 1.17), and PTG6 (“New path for life”) (EI value: 0.92) in the network. These findings align with the results observed in the individual network models for PTS and PTG, as depicted in Supplementary Figure S1. Regarding BEI centrality, PTS3 (“Avoidance of thoughts”) within the PTS cluster exhibited the highest positive BEI (BEI value: 1.68), while PTG9 (“Perception of greater personal strength”) in the PTG cluster showed the highest negative BEI (BEI value: -2.21). The EI and BEI values for each node are illustrated in Fig. 2.

### Network stability and accuracy

**Fig. 3:**
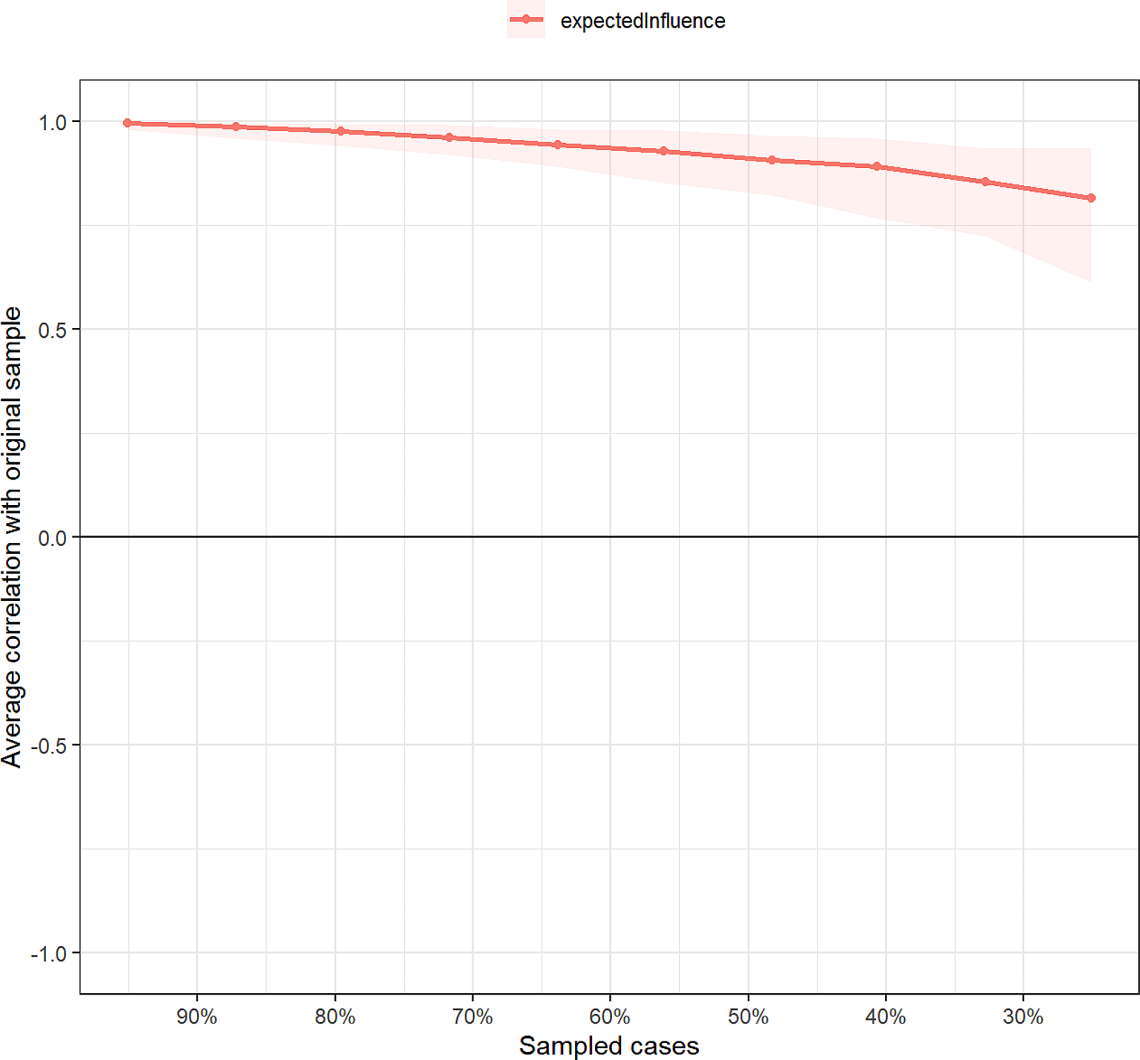
Stability of expected influence indices using case-dropping bootstrap

Figure 3 shows the results of the case-dropping bootstrap test. In terms of network stability, the CS coefficient of centrality EI was 0.67 (95% CI: 0.59–0.75), suggesting that 67% of the nodes in the sample could be randomly dropped without significantly changing the network structure. The bootstrapped 95% CIs for most edge weights were relatively narrow, indicating an accurate network structure (see Supplementary Figure S2). Additionally, nonparametric bootstrapped difference tests revealed significant differences among most edge weights and node EIs (see Supplementary Figure S3 and S4).

## Discussion

To the best of our knowledge, this study represents the first attempt to apply network analysis to examine the co-occurring patterns of PTS and PTG in pregnant women who have experienced pregnancy loss. In the network model, the PTS symptoms and PTG symptoms displayed visually distinct clusters, along with shared positive and negative connections, consistent with findings from previous network analysis studies [16, 17].

Our findings revealed the central role of avoidance symptoms within the network, as evidenced by their strong edge weights and high centrality. Consistent with our research, women with a history of traumatic childbirth often employ avoidance as a psychological defense mechanism, attempting to sidestep directly confronting distressing memories and emotions associated with the experience [24]. Moreover, prior experiences of pregnancy loss may cause women to feel extremely anxious about their current pregnancy or childbirth, and avoiding thoughts related to pregnancy loss could serve as a means of protecting oneself from triggering these anxious emotions and seeking psychological shelter [25]. Regarding PTG symptoms, the most influential aspect is the transformation of new possibilities, aligning with the majority of findings in current research on PTG network analysis [13, 14, 26]. In studies related to pregnancy loss, participants also reported experiencing personal growth by adapting to their needs, cultivating new interests, and engaging in charitable volunteer work [27]. In the realm of network theory, alterations in central symptoms can significantly impact other symptoms within the model [11]. Therefore, guiding pregnant individuals who have experienced pregnancy loss to shift their focus towards new possibilities can assist them in better adapting to their circumstances and achieving personal growth.

The observed bridge symptoms play a crucial role in comprehending the shared psychopathological structure of PTS and PTG [23]. In this study, for the sample of pregnant women, avoidance of traumatic thoughts related to prior pregnancy loss was found to activate the PTG symptom cluster. Although prior research has predominantly suggested that avoidance coping hinders adaptation to loss and inhibits PTG, some findings have indicated that short-term avoidance does not necessarily imply a negative avoidance strategy but rather signifies a flexible “adaptive avoidance” [28]. Actively redirecting attention from past painful loss experiences to the current hopeful pregnancy, refraining from dwelling on the past, and adopting a future-oriented coping approach seem to promote positive cognition and psychological transformations in pregnant women [6, 27]. Moreover, it is intriguing to note that within the PTG cluster, perceiving greater personal strength can suppress the PTS symptom network, particularly when it exhibits a strong negative correlation with intrusive rumination. Qualitative research has demonstrated that some women, following a pregnancy loss event, discover their resilience and realize that their inner strength aids them in actively countering negative emotions and reducing intrusive thoughts [27]. Consequently, it is essential to explore methods for enhancing self-efficacy (i.e., confidence in coping with difficulties) in pregnant women with a history of pregnancy loss to alleviate their PTS symptoms.

There are some limitations that should be acknowledged. First, due to the cross-sectional study design, we could not assess the causal relationship and dynamic changes related to the association between PTS symptoms and PTG symptoms. Second, although both the IES-6 and PTGI-SF scales have good psychometric properties in Chinese populations [19, 21], the use of simplified scales for measuring PTS and PTG symptoms may limit the comprehensiveness of symptom assessment. Third, our investigation was conducted during the COVID-19 pandemic, a period in which the post-traumatic psychological well-being of pregnant women may be influenced by the stress of contracting the virus. Fourth, this study focused on pregnant women in the early stages of pregnancy, thereby restricting the generalizability of our findings to pregnant women in different phases of gestation. Finally, while the sample size in this study is sufficient for network analysis [22], it is not adequate to support network comparison tests among different subgroups. Future research should expand the sample size to more comprehensively explore the differences in the co-occurrence networks of PTS and PTG among various samples.

## Conclusion

In summary, this study identifies the central symptoms in the co-occurrence network of PTS and PTG as “Avoidance of thoughts,” “Ability to do better things,” and “New path for life.” The bridge nodes connecting PTS and PTG are “Avoidance of thoughts” and “Perception of greater personal strength.” This provides a theoretical foundation for targeted interventions aimed at alleviating the severity of PTS among pregnant women with a history of pregnancy loss in the future and promoting their personal growth.

### Electronic supplementary material

Below is the link to the electronic supplementary material.

Supplementary Material 1

## Data Availability

The datasets used and/or analyzed during the current study are available from the corresponding author upon reasonable request.

## Abbreviations

PTS: Posttraumatic stress
PTG: Posttraumatic growth
IES-6: Impact of Events Scale-6
PTGI-SF: Posttraumatic Growth Inventory-Short Form
