# Supplementary material for: Network analysis of posttraumatic stress and posttraumatic growth symptoms among women in subsequent pregnancies following pregnancy loss
Source: BMC Psychiatry. 2024 Apr 9;24:266. doi: 10.1186/s12888-024-05702-6 (PMC11003179; doi:10.1186/s12888-024-05702-6)
Supplement: Supplementary file 1 — Supplementary Material 1 [file 12888_2024_5702_MOESM1_ESM.docx]

Table S1. Mean and standard deviation of the PTS and PTG items.

| **Symptoms** | **Mean** | **SD** |
| --- | --- | --- |
| **PTS** |  |  |
| PTS1: Triggered memories | 1.42 | 0.97 |
| PTS2: Intrusive rumination | 1.60 | 1.08 |
| PTS3: Avoidance of thoughts | 1.05 | 0.84 |
| PTS4: Avoidance of feelings | 0.90 | 0.85 |
| PTS5: Concentration problems | 0.89 | 0.82 |
| PTS6: Hypervigilance | 0.78 | 0.82 |
| **PTG** |  |  |
| PTG1: Changed priorities | 2.99 | 1.33 |
| PTG2: Greater appreciation for life | 2.93 | 1.19 |
| PTG3: Ability to do better things | 3.61 | 1.19 |
| PTG4: Better understanding of spiritual matters | 3.24 | 1.32 |
| PTG5: Greater interpersonal closeness | 3.49 | 1.11 |
| PTG6: New path for life | 3.24 | 1.17 |
| PTG7: Ability to handle difficulties | 3.05 | 1.46 |
| PTG8: Stronger religious faith | 3.05 | 1.25 |
| PTG9: Perception of greater personal strength | 3.27 | 1.47 |
| PTG10: Wonderful people | 3.41 | 1.16 |

Table S2. Correlation matrix of the PTS and PTG items.

| **Items** | **PTS1** | **PTS2** | **PTS3** | **PTS4** | **PTS5** | **PTS6** | **PTG1** | **PTG2** | **PTG3** | **PTG4** | **PTG5** | **PTG6** | **PTG7** | **PTG8** | **PTG9** | **PTG10** |
| --- | --- | --- | --- | --- | --- | --- | --- | --- | --- | --- | --- | --- | --- | --- | --- | --- |
| **PTS1** | 0.000 | 0.383 | 0.000 | 0.151 | 0.129 | -0.054 | 0.031 | -0.054 | 0.000 | 0.000 | 0.065 | 0.000 | 0.000 | -0.071 | -0.090 | 0.000 |
| **PTS2** | 0.383 | 0.000 | 0.230 | 0.197 | -0.084 | 0.000 | 0.009 | 0.089 | 0.055 | 0.012 | 0.000 | 0.000 | -0.090 | 0.107 | -0.247 | 0.057 |
| **PTS3** | 0.000 | 0.230 | 0.000 | 0.563 | 0.176 | 0.024 | 0.000 | 0.000 | 0.020 | 0.000 | 0.000 | -0.047 | 0.184 | -0.017 | 0.000 | 0.000 |
| **PTS4** | 0.151 | 0.197 | 0.563 | 0.000 | 0.044 | 0.130 | 0.000 | -0.093 | 0.000 | 0.121 | -0.065 | 0.030 | 0.000 | 0.000 | 0.010 | -0.052 |
| **PTS5** | 0.129 | -0.084 | 0.176 | 0.044 | 0.000 | 0.510 | 0.091 | 0.000 | -0.135 | -0.003 | 0.000 | -0.014 | -0.105 | -0.037 | 0.157 | 0.152 |
| **PTS6** | -0.054 | 0.000 | 0.024 | 0.130 | 0.510 | 0.000 | -0.053 | 0.071 | 0.077 | -0.128 | 0.000 | 0.000 | 0.046 | 0.017 | -0.032 | -0.134 |
| **PTG1** | 0.031 | 0.009 | 0.000 | 0.000 | 0.091 | -0.053 | 0.000 | 0.475 | 0.019 | 0.000 | -0.073 | 0.353 | -0.054 | 0.000 | 0.000 | 0.149 |
| **PTG2** | -0.054 | 0.089 | 0.000 | -0.093 | 0.000 | 0.071 | 0.475 | 0.000 | 0.103 | 0.194 | 0.000 | 0.019 | 0.000 | 0.157 | -0.141 | 0.000 |
| **PTG3** | 0.000 | 0.055 | 0.020 | 0.000 | -0.135 | 0.077 | 0.019 | 0.103 | 0.000 | 0.378 | 0.085 | 0.054 | 0.105 | 0.000 | 0.391 | 0.195 |
| **PTG4** | 0.000 | 0.012 | 0.000 | 0.121 | -0.003 | -0.128 | 0.000 | 0.194 | 0.378 | 0.000 | 0.219 | 0.041 | 0.000 | 0.071 | 0.000 | -0.132 |
| **PTG5** | 0.065 | 0.000 | 0.000 | -0.065 | 0.000 | 0.000 | -0.073 | 0.000 | 0.085 | 0.219 | 0.000 | 0.023 | 0.000 | 0.377 | 0.000 | 0.330 |
| **PTG6** | 0.000 | 0.000 | -0.047 | 0.030 | -0.014 | 0.000 | 0.353 | 0.019 | 0.054 | 0.041 | 0.023 | 0.000 | 0.206 | 0.264 | 0.036 | 0.107 |
| **PTG7** | 0.000 | -0.090 | 0.184 | 0.000 | -0.105 | 0.046 | -0.054 | 0.000 | 0.105 | 0.000 | 0.000 | 0.206 | 0.000 | -0.043 | 0.460 | -0.119 |
| **PTG8** | -0.071 | 0.107 | -0.017 | 0.000 | -0.037 | 0.017 | 0.000 | 0.157 | 0.000 | 0.071 | 0.377 | 0.264 | -0.043 | 0.000 | -0.026 | 0.163 |
| **PTG9** | -0.090 | -0.247 | 0.000 | 0.010 | 0.157 | -0.032 | 0.000 | -0.141 | 0.391 | 0.000 | 0.000 | 0.036 | 0.460 | -0.026 | 0.000 | 0.114 |
| **PTG10** | 0.000 | 0.057 | 0.000 | -0.052 | 0.152 | -0.134 | 0.149 | 0.000 | 0.195 | -0.132 | 0.330 | 0.107 | -0.119 | 0.163 | 0.114 | 0.000 |


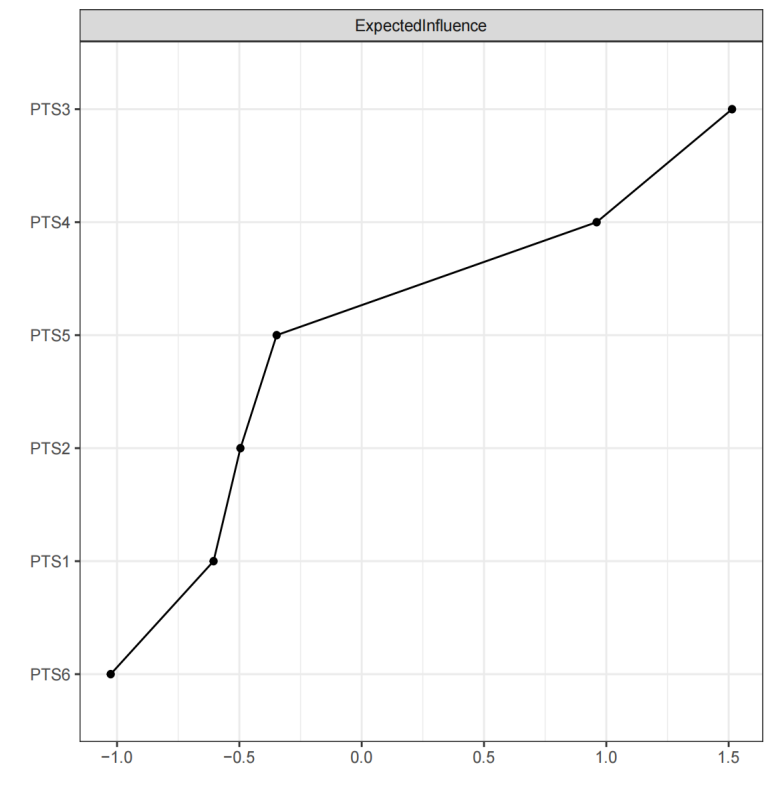

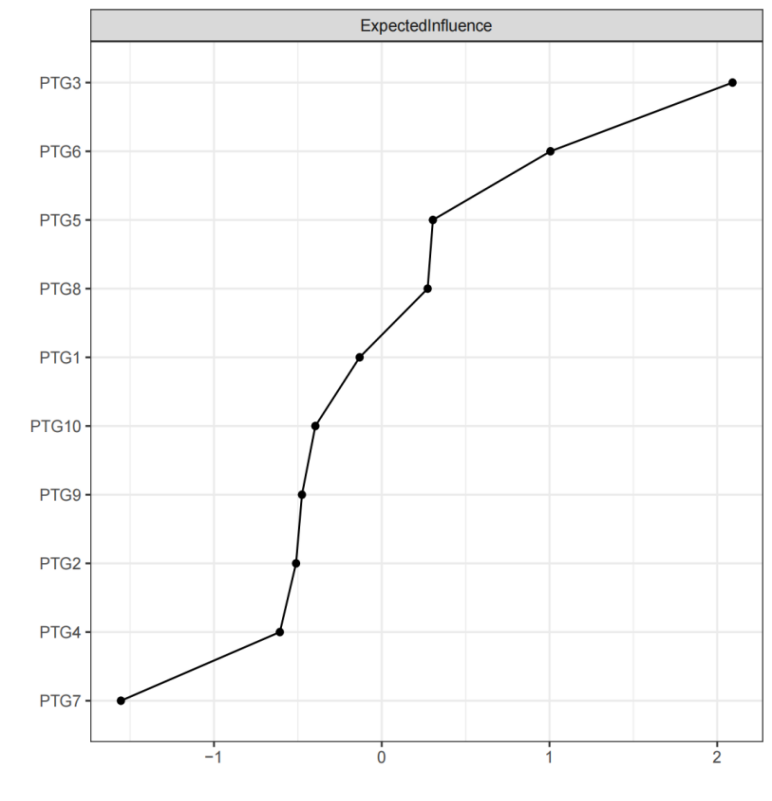


Figure S1. Expected influence of PTS and PTG symptoms within their respective network structures.


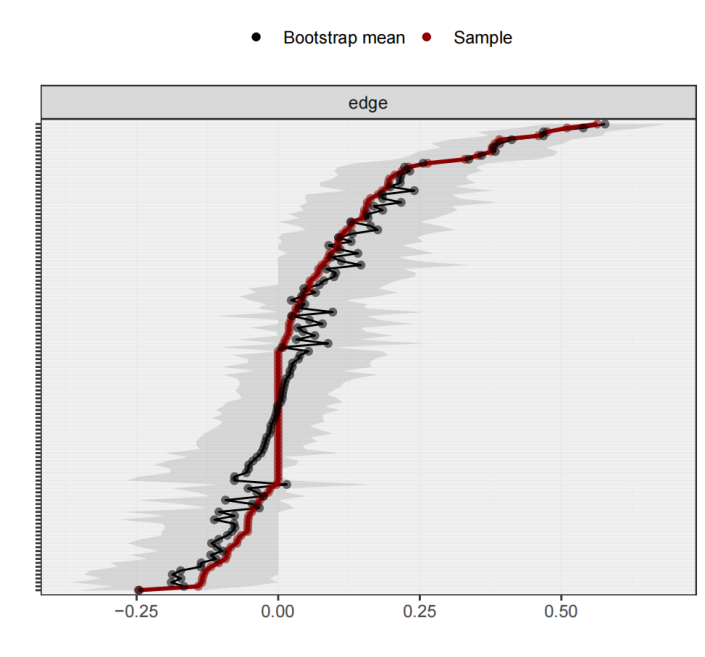


Figure S2. The accuracy of network edges by non-parametric bootstrapping.

Nodes: The gray area represents the bootstrap 95% confidence interval.


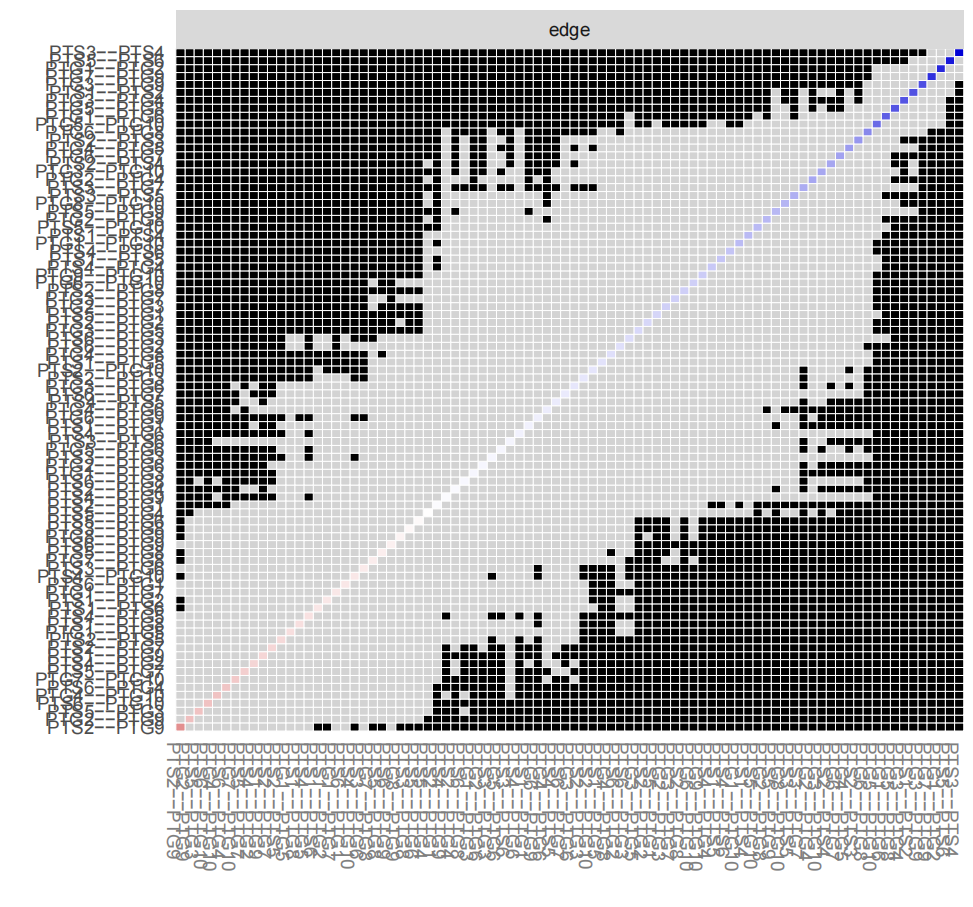


Figure S3. Tests of significant differences between edge weights in the network.

Nodes: Black boxes indicate a significant difference between edge weights, while grey boxes indicate no significant difference.


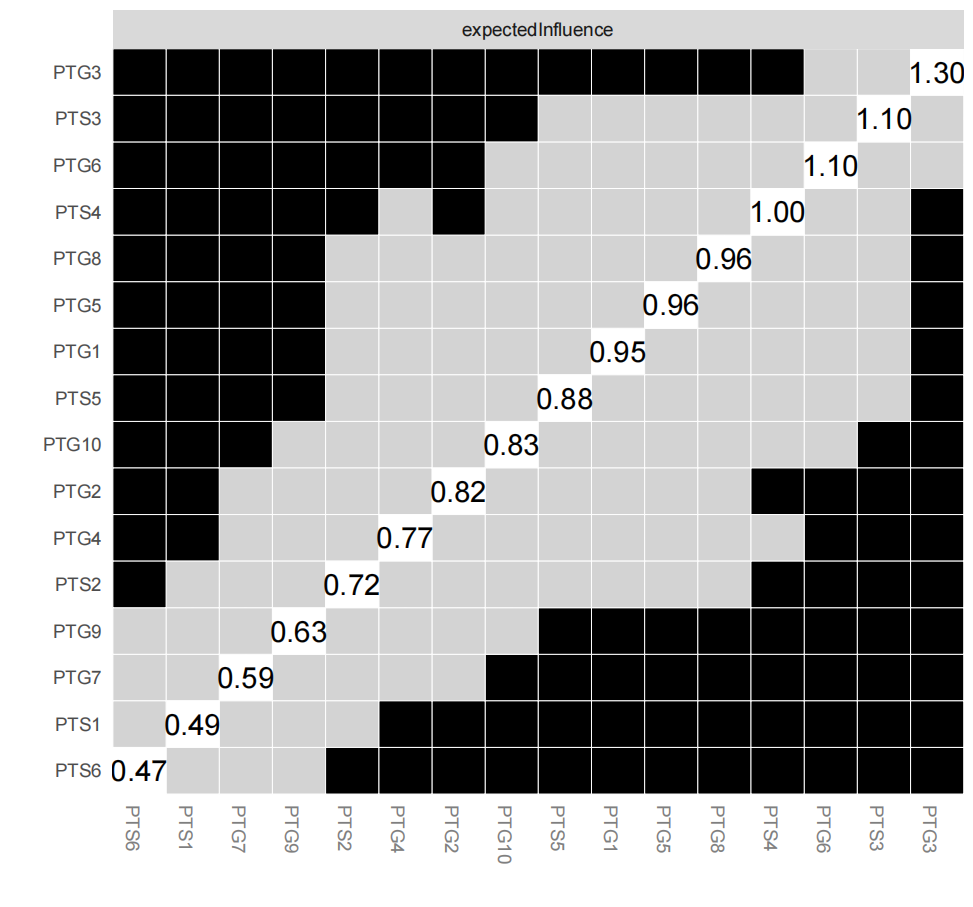


Figure S4. Tests of significant differences between expected influence values of nodes in the network.

Nodes: Black boxes indicate a significant difference between expected influence values of nodes, while grey boxes indicate no significant difference.
